# Ontogeny of drug-induced fatty liver disease (DIFLD): from key initiating events to disease phenotypes

**DOI:** 10.1007/s00204-025-04178-x

**Published:** 2025-09-16

**Authors:** Ernesto López-Pascual, Marta Moreno-Torres, Erika Moro, Anna Rapisarda, Rita Ortega-Vallbona, Eva Serrano-Candelas, Rafael Gozalbes, Ramiro Jover, José V. Castell

**Affiliations:** 1 Department of Biochemistry and Molecular Biology. Faculty of Medicine, University of Valencia, Valencia, Spain; 2 Unit for Experimental Hepatology, Health Research Institute La Fe (IISLAFE), Valencia, Spain; 3 Centro de Investigación Biomédica en Red de Enfermedades Hepáticas y Digestivas (CIBERehd), Instituto de Salud Carlos III, Madrid, Spain; 4 ProtoQSAR SL., Centro Europeo de Empresas e Innovación (CEEI), Parque Tecnológico de Valencia, Av. Benjamín Franklin, 12, 46980 Paterna, Valencia Spain

**Keywords:** Drug-induced fatty liver disease (DIFLD), Steatosis, Steatohepatitis, Mitochondrial dysfunction, Physicochemical properties, Toxicity mechanisms

## Abstract

We conducted an expert review of clinical case reports on drug-induced fatty liver disease (DIFLD) to classify drugs according to distinct clinical phenotypes. Seven clusters were identified based on clinical, biochemical, and histological features reflecting drug toxic mechanisms:

- Cluster 0 (Control): Drugs with no known steatotic effects or clinical evidence of DIFLD.
- Cluster 1: Drugs with mild pro-steatotic effects, exacerbating existing metabolic steatosis without significant liver enzyme elevation.
- Cluster 2: Compounds causing moderate steatosis with mild hepatocellular damage, occasional enzyme increases, and delayed onset.
- Cluster 3: Agents causing severe mitochondrial dysfunction, ATP depletion, and lactic acidosis, initially without inflammation.
- Cluster 4: Drugs inducing inflammatory steatohepatitis with moderate elevations of liver enzymes (ALT, AST, ALP 90–700 U/L) but preserved liver function.
- Cluster 5: Drugs causing severe steatohepatitis with marked enzyme elevation (ALT, AST > 700 U/L) indicating significant liver injury and inflammation.
- Cluster 6: Compounds causing steatohepatitis with additional cholestasis and elevated bilirubin (> 11 mg/dL).

Clusters 1 and 2 primarily impair β-oxidation and mitochondrial respiration, linked to high lipophilicity and typically lower daily doses. Cluster 3 involves mitochondrial DNA depletion and impaired lipid export. Clusters 4 and 5 combine mitochondrial and nuclear receptor disruption, often linked to higher daily doses. Cluster 6 combines steatosis-promoting mechanisms with bile acid transport disruption. This classification improves understanding of DIFLD phenotypes by linking clinical manifestations with drug physicochemical properties and toxicological mechanisms, aiding diagnosis and risk assessment of drug-induced steatosis.

**Supplementary Information:**

The online version contains supplementary material available at 10.1007/s00204-025-04178-x.

## Introduction

Drug-induced fatty liver disease (DIFLD) is a specific form of drug-induced liver injury (DILI), and is classified under the umbrella term Steatotic Liver Disease (SLD), which also includes metabolic dysfunction-associated steatotic liver disease (MASLD). MASLD refers to the accumulation of fat in the liver by metabolic dysfunction, unrelated to alcohol consumption (formerly NAFLD), and DIFLD represents another branch of the generic umbrella term SLD (Rinella et al. 2023). DIFLD encompasses various biochemical and histopathological manifestations, including macrovesicular and microvesicular steatosis, mitochondrial dysfunction, liver inflammation (steatohepatitis), and further fibrosis (Kolaric et al. 2021; López-Pascual et al. 2024). The spectrum of clinical presentation in DIFLD is diverse, ranging in nature, extent, and severity of liver injury (Kleiner et al. 2014; Saithanyamurthi and Faust 2017).

Steatosis has traditionally been diagnosed and graded through liver biopsy, based on criteria, such as lipid droplet size, the proportion of parenchyma affected by steatosis, and the overall extent of liver tissue involved. Modern classification systems now use a semiquantitative scale (S0–S3) to indicate the magnitude and severity of fat accumulation (Kleiner et al. 2005). S0 represents the absence of steatosis, with a normal liver and no significant fat accumulation in hepatocytes. S1 indicates mild steatosis, with fat accumulation present in 5–33% of hepatocytes. S2 reflects moderate steatosis, affecting 34–66% of hepatocytes, while S3 denotes severe steatosis, with more than 66% of hepatocytes involved. However, this classification does not consider the disease phenotype or the underlying mechanisms contributing to steatosis.

Through an extensive review of clinical case reports and published literature, we have identified and classified representative drugs associated with distinct phenotypes of drug-induced fatty liver disease (DIFLD). Each phenotype exhibits specific analytical, clinical, and morphological characteristics. Similar to non-alcoholic fatty liver disease (NAFLD), the clinical spectrum of DIFLD phenotypes ranges from mild, asymptomatic hepatic steatosis to more severe pathological conditions and liver dysfunction (Sanyal et al. 2021). Indeed, in certain individuals, an initial drug-induced steatosis event can be exacerbated by additional injury mechanisms, such as oxidative stress, resulting in hepatocyte death and inflammation (DISH). This progression shifts the condition from simple steatosis (DIS) to drug-induced steatohepatitis (DISH), a form of drug induced liver injury (DILI) characterized by intracellular accumulation of lipids in hepatocytes accompanied by subsequent inflammatory (Satapathy et al. 2015). DISH is associated with a significantly worse clinical prognosis, as it entails persistent hepatic inflammation and, in some cases, progression to fibrosis, ultimately resulting in more severe liver dysfunction (Dash et al. 2017).

To better understand the complexity of DIFLD, we have thoroughly examined the clinical signs, laboratory data, and liver biopsy results of unequivocally diagnosed patients. In parallel, we then examined the underlying mechanisms by which the drugs cause the liver injury. Several mechanisms seem to be involved, as previously reviewed by us (López-Pascual et al. 2024). In addition, we have sought to identify molecular and physicochemical features of the offending drugs that may predispose them to cause the different DIFLD phenotypes. The structural characteristics of these compounds, which allowed their classification as potential inducers of drug-induced fatty liver (DIFL), may also serve as predictive markers for anticipating the hepatotoxic risk in other, as yet uncharacterized, pharmacological agents.

Our investigation highlights compelling evidence that certain pre-existing conditions can significantly predispose individuals to drug-induced fatty liver disease (DIFLD). These comorbidities not only heighten hepatic vulnerability to drugs typically regarded as non-steatogenic or only mildly pro-steatotic, but may also facilitate the transition from benign steatosis to more advanced and harmful forms of liver injury. In particular, coexisting metabolic disorders, such as obesity type 2 diabetes and dyslipidemia, appear to act as critical amplifiers of this condition, underscoring the importance of considering patient-specific risk factors in the assessment of the hepatic safety profile of a given drug.

Given the marked clinical heterogeneity of DIFLD, early identification of disease phenotypes and their underlying mechanisms is critical for anticipating and preventing the progression of liver injury. Routine monitoring of liver function in patients receiving potentially hepatotoxic drugs is especially important in those with predisposing risk factors. Further research into the molecular drivers of DIFLD, along with the development of predictive biomarkers, holds promise for improving patient stratification and informing targeted clinical management strategies (Andrade et al. 2019). Moreover, a deeper understanding of the structural features of drugs that contribute to DIFLD may guide the rational design of safer therapeutic agents. In this context, this study provides an integrative framework linking drug properties, host factors, and phenotypic manifestations, thereby advancing the predictive and preventive landscape of drug-induced fatty liver disease.

## Materials and methods

### Scientific literature search strategy

To compile a database of steatogenic compounds and associated patient clinical features, we first conducted an extensive search of drug-induced steatosis cases in PubMed and LiverTox, which identified a series of compounds frequently implicated in DIFLD. We then designed a literature search strategy to identify clinical cases and reports, using key terms covering the compound of interest, its drug class, fatty liver disease, hepatotoxicity, and other relevant clinical features. In addition, specific terms were included to restrict the search to this type of publication (clinical cases or reports), following the Boolean search structure: ((chemical name [Title] OR synonyms [Title]) OR (drug class [Title] AND (chemical name OR synonyms))) AND (Fatty liver-related terms OR hepatotoxicity-related terms) AND case report-related terms.

The search was conducted without time restrictions to maximize the inclusion of relevant records. A total of 14,115 articles were retrieved and subsequently curated.

### Artificial intelligence-based article selection and data extraction

#### Case reports selection with artificial intelligence support and validation of the AI model

Sysrev (Bozada et al. 2021), a web-based platform powered by artificial intelligence that allows users to upload and evaluate documents, configure review workflows, invite collaborators, and streamline the screening process, was employed to examine titles and abstracts obtained from the search strategy output, with the aim of identifying relevant clinical case reports. Sysrev’s artificial intelligence system applies an active learning strategy to iteratively train neural network-based models for document screening. Once an initial subset of articles has been evaluated by users, the system dynamically reprioritizes the remaining documents to optimize the efficiency of model training. Sysrev currently incorporates LLM-based models alongside other artificial intelligence tools. For this study, the active learning module was employed to streamline the inclusion/exclusion decision process. Following manual classification of 1412 titles and abstracts as either included or excluded, the AI-assisted auto-labelling feature was activated. The model achieved a recall of 92%, a precision of 85%, a specificity of 90%, and an overall accuracy of 91% and the model was subsequently applied to the rest of the data set. Ultimately, 791 case reports deemed both accessible and potentially informative were selected, and their full-text PDFs were uploaded to Sysrev for structured data extraction and review.

#### Data extraction and curation

Accessible PDF uploaded to Sysrev were expert reviewed to exclude those useless for our purpose, missing essential clinical details, those where hepatic steatosis was not verified through liver biopsy or imaging methods (e.g., MRI or ultrasound), or where the causal relationship between the drug and the steatotic event was ambiguous or not well-supported. Clinical information was extracted for each report, including laboratory test results, such as alkaline phosphatase (ALP), alanine aminotransferase (ALT), aspartate aminotransferase (AST), bilirubin levels, and coagulation markers, along with imaging and biopsy-derived findings. In addition, relevant comorbidities (e.g., obesity, diabetes, and alcohol use) were collected. Data curation and normalization of biochemical variables not originally provided in standardized measurement units were performed essentially as described in our previous study, which followed a similar methodological approach in the context of cholestasis (Moreno-Torres et al. 2024).

Case reports involving compounds outside the scope of this study were excluded from the analysis. These included industrial or environmental chemicals (e.g., 1,1,1-trichloroethane and vinyl chloride), protein-based agents (e.g., asparaginase), and widely used drugs for which there is minimal or no clinical evidence linking them to hepatic steatosis despite their broad administration. For instance, ibuprofen was excluded, as only two rare and atypical cases were reported (Gadaleta et al. 2024). Finally, the clinical and histopathological data derived from 217 case reports drawn from 147 articles were used for the purpose of this study. These data are publicly available in the BioStudies repository under accession number S-ONTX76.

### Data compilation and QSAR modelling of physicochemical and pharmacokinetic properties of steatogenic drugs

Chemical structures in SMILES format were obtained from the PubChem database (https://pubchem.ncbi.nlm.nih.gov/) using the corresponding ChEMBL identifiers. The SMILES were preprocessed using the RDKit Python library (https://www.rdkit.org/) to remove counterions and standardize molecular structures. Various QSAR models were then applied to best estimate key physicochemical and ADME properties, following the general methodology outlined by Moreno-Torres et al. (2024) with some minor modifications. More precisely, pKa-a and pKa-b values were predicted using the OPERA tool (https://ntp.niehs.nih.gov/whatwestudy/niceatm/comptox/ct-opera/opera) as the first-choice method and ADMETLab 3.0 (https://admetlab3.scbdd.com/) as an alternative. ADMETLab 3.0 was also employed as a secondary predictor for the boiling point (BP). For melting point (MP), ProtoPRED (https://protopred.protoqsar.com/) served as a secondary tool, while it was used as the primary predictor for logD and F30 values. Predictions for logH and Caco-2 permeability were made using OPERA as the first choice and ADMETLab 3.0 as a backup. Finally, VNN–ADMET was utilized as the primary model for predicting P-glycoprotein (P-gp) substrate affinity. When available, experimental values were preferred over predicted estimates. Tool prioritisation was performed based on the results described in Gadaleta et al. (2024) but using OPERA and ADMETLab3 instead of ChemAxon for pKa-a and pKa-b prediction, because they are free of charge.

### Statistical and cluster analysis

A hierarchical clustering dendrogram was constructed using MetaboAnalyst (https://dev.metaboanalyst.ca/), (Pang et al. 2021) based on categorized median values and incidence frequencies of selected clinical and histopathological variables per compound, as follows: ALT median values from 40 to 90 U/L were assigned a value of 1; 90 to 700, a value of 2; 700 to 1400, a value of 3; and values above 1400, a value of 4. Total bilirubin levels from 1.2 to 2 mg/dL were assigned a value of 1; 2 to 5, a value of 2; 5 to 11, a value of 3; and above 11, a value of 4. Lactic acid levels between 30 and 50% incidence were assigned a value of 1, and those above 50%, a value of 2. Similarly, duct injury incidence between 30 and 50% were assigned a value of 1, and those above 50%, a value of 2. The Euclidean distance metric was used to calculate dissimilarity, and the Ward linkage method was applied to define cluster structure.

## Results

### DIFL phenotypes and the clustering of drugs, based on clinical data features

**Fig. 1:**
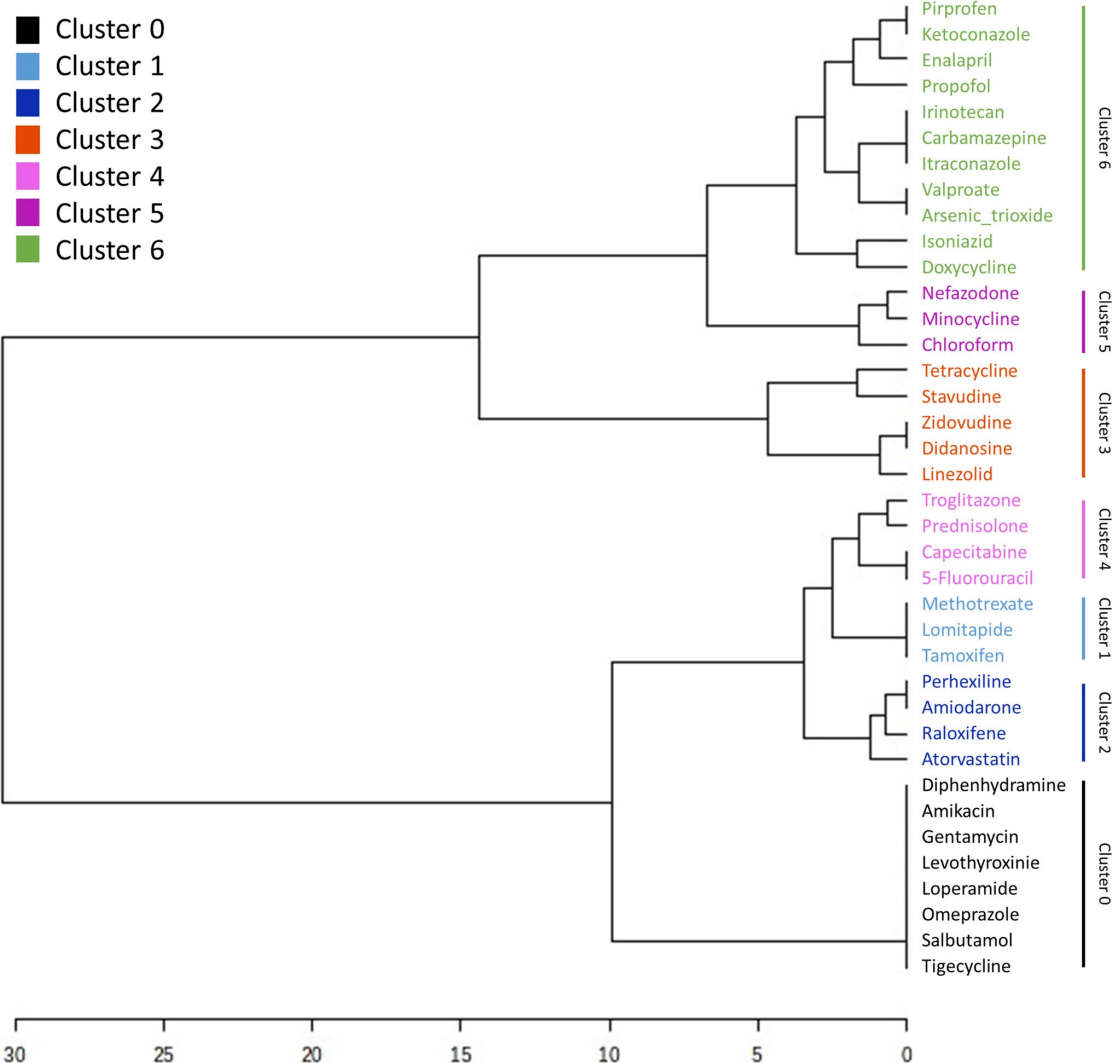
Hierarchical clustering of drugs based on clinical and histopathological features from case reports. Hierarchical dendrogram showing the clustering of drugs based on the median or frequency of clinical laboratory values, and histopathological data of case reports associated with each drug taken from Table 1

To better understand the clinical diversity of drug-induced fatty liver disease (DIFLD), we undertook a systematic analysis of reported cases to identify distinct phenotypic patterns. We first analysed the data set extracted from literature DIFLD case reports, guided by expert insights aimed at uncovering meaningful patterns of DIFLD disease phenotypes (Table S1). Furthermore, stratification of the retrieved clinical parameters was performed by applying predefined cutoffs to delineate potential disease phenotype clusters. Thereafter, we constructed a dendrogram summarizing these findings to visualize the relationships among them (Fig. 1). It then emerged that only a few parameters were most determinant and discriminative among the different phenotype clusters, as summarized in Table 1. This hierarchical clustering approach, supported by clinical data, revealed distinct clusters corresponding to different disease subtypes, providing valuable insight into the diversity of clinical manifestations:

**Table 1:**
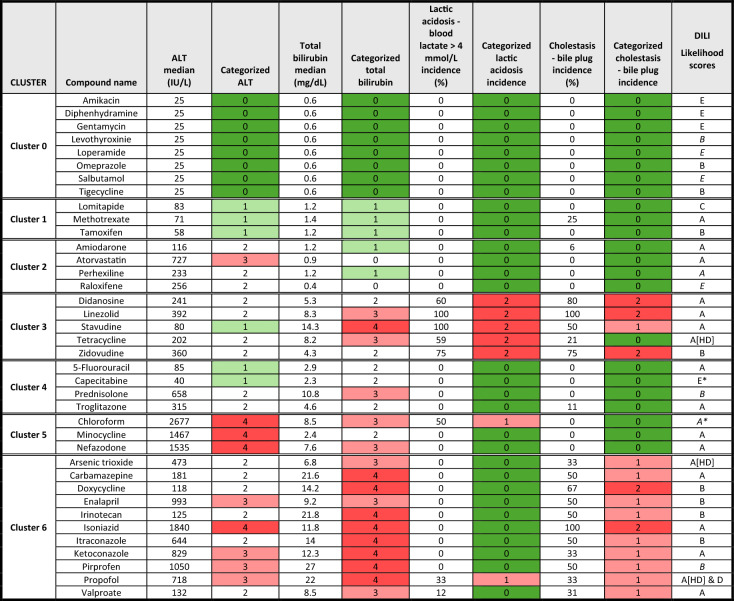
Drugs and discriminating parameters used in the hierarchical clustering analysis

Hierarchical clustering dendrogram showing the clustering of drugs based on the median or frequency values derived from clinical laboratory values, and histopathological results from the case reports associated with each drug, as detailed in Table 1. This clustering approach allowed the identification of drug clusters that display similar clinical and histopathological disease patterns in DIFLD-affected patients (Table S1). Cluster 0 includes compounds with no known or reported steatotic potential. Cluster 1 contains drugs with mild pro-steatotic activity, often showing subtle metabolic effects. Cluster 2 includes agents causing moderate steatosis with signs of metabolic disruption. Cluster 3 is characterized by severe mitochondrial dysfunction and lactic acidosis. Cluster 4 comprises compounds associated with significant biochemical liver alterations. Cluster 5 includes drugs linked to steatohepatitis with markedly elevated hepatic enzymes. Cluster 6 is defined by drugs inducing steatohepatitis with cholestatic features, such as bile duct injury and elevated bilirubin.

Table 1 displays the drugs and the most discriminating clinical and histopathological parameters used to construct the hierarchical cluster analysis depicted in Fig. 1. For each drug, the median value or percentage of incidence observed in related case reports is shown, alongside the corresponding categorized value used in the clustering dendrogram. Clinical variables were categorized prior to clustering to enable comparative scaling across drugs, as described in the Materials and Methods section. A green-to-red color gradient was used to highlight values for each categorized variable, with the highest values shown in red and the lowest in green. Cluster 0 includes reference to non-steatogenic compounds, characterized by normal clinical parameters. Cluster 1 shows mild alanine aminotransferase (ALT) elevations with bilirubin near the normal range and no evidence of lactic acidosis or bile accumulation. Cluster 2 is characterized by moderate increases in ALT and occasional bilirubin elevation, but without systemic acidosis or cholestasis. Cluster 3 displays moderate elevations in liver enzymes and is primarily defined by the presence of lactic acidosis and occasionally signs of cholestasis, with bilirubin levels within a mild range. Cluster 4 is defined by increased ALT, mild increases in bilirubin and no evidence of lactic acidosis or cholestasis. Cluster 5 includes drugs causing severe steatohepatitis with marked enzyme elevation, indicating significant liver injury and inflammation. Cluster 6 contains compounds causing steatohepatitis with additional cholestasis and elevated bilirubin. The “DILI Likelihood score” gathers categorization of the likelihood of drug induced liver injury (DILI) information extracted from the LiverTox database (LiverTox 2019b) or inferred, based on the Food and Drug Administration (FDA) DILIrank classification (FDA 2023). The categories are: A, well-established either direct or idiosyncratic liver injury; B, likely to cause idiosyncratic injury; C, probable linked to idiosyncratic liver injury; D, possible hepatotoxin; E*, suspected but unconfirmed; E, not believed or unlikely to cause liver injury; HD, only hepatotoxic at high doses. When data were not recorded in LiverTox, estimated scores (in italics) were inferred from the U.S. Food and Drug Administration (FDA) DILIrank classification as follows: “Most” DILI-Concern was mapped to A, “Less” to category B, “Ambiguous” to category E* and “No” to category E. A* refers to the case of chloroform, which was not described in either database but is known to produce steatosis (Table 1).

#### Cluster 0: Drugs with no known or reported Steatotic Potential

Drugs in this cluster have no known steatotic potential in humans. Despite being widely used, no clinical reports have been found in the literature indicating these drugs alter hepatic functionality and cause steatosis. Clinical parameters in patients using such drugs are within normal reference values.

#### Cluster 1: Drugs with mild pro-steatotic potential DIFL

This category includes drugs that possess a mild capacity to promote hepatic steatosis. These drugs are likely to exacerbate pre-existing metabolic conditions rather than directly induce liver damage, as there are few reported cases of DIFLD associated with their use in patients without prior liver disease. These agents do not usually cause significant elevations in biochemical markers of hepatocellular injury, such as ALT, AST, GGT, or bilirubin. Steatosis associated with these drugs tends to develop gradually, often requiring prolonged exposure before clinical signs become evident, and occasionally progress to fibrosis (Tables 1 and S1).

Consequently, the risk of drug-induced steatosis in this cluster is significantly influenced by predisposing factors, particularly diabetes, obesity and alcohol consumption, which can intensify drug-induced metabolic imbalances. The hallmark pathological feature is the presence of macrovesicular steatosis, characterized by the accumulation of large lipid droplets within hepatocytes, without severe metabolic dysfunction.

*Methotrexate* and *tamoxifen* are typical examples of drugs in this category. Both have been associated with cases of hepatic steatosis, particularly in individuals with pre-existing metabolic conditions (Meunier and Larrey 2020; Agoglia et al. 2021). However, despite their pro-steatotic potential, the absence of severe hepatic injury in most cases suggests that these agents primarily worsen already compromised lipid metabolism rather than directly provoking the liver damage.

#### Cluster 2: Drugs causing moderate steatosis with noticeable metabolic disturbance

Drugs in this cluster are associated with the development of moderate (macro-, micro-) steatosis, characterized by a more noticeable disruption in metabolic function compared to Cluster 1. The clinical phenotype is notable for large time to onset of steatosis, with patients often presenting with more significant biochemical alterations, such as mild-to-moderate elevations in liver enzymes (e.g., ALT and AST) and bilirubin. While the degree of hepatocyte damage is typically mild, inflammatory changes and fibrosis may occur, in some cases, at later stages of disease.

Unlike the mild steatosis seen in Cluster 1, the metabolic disturbance in Cluster 2 is more substantial, and these biochemical changes may serve as early indicators of hepatocellular injury. The condition is more likely to progress toward inflammation, fibrosis, and other histopathological changes over time, especially in patients with pre-existing and consolidated metabolic conditions such as diabetes. The presence of these comorbidities significantly accelerates disease progression and more severe liver involvement.

Drugs in this cluster may predispose susceptible individuals to a worsening of liver function, with the potential for the steatosis to evolve into steatohepatitis or other forms of chronic liver disease if not adequately managed.

#### Cluster 3: Severe mitochondrial damage and dysfunction causing lactic acidosis

This cluster of drugs is primarily characterized by their association with lactic acidosis, a severe metabolic complication within the spectrum of drug-induced liver injury, particularly in cases involving steatosis. The underlying mechanism involves mitochondrial dysfunction and ATP depletion, which trigger a compensatory upregulation of glycolysis, leading to excessive lactate production and culminating in lactic acidosis (LiverTox 2019a). It is frequently accompanied by microvesicular steatosis in the hepatocytes. Histologically, microvesicular steatosis represents the accumulation of numerous small lipid droplets within hepatocytes, reflecting the mitochondrial dysfunction's impact on lipid metabolism. Microvesicular steatosis is typically associated with impaired mitochondrial fatty acid β-oxidation and dysfunction. In contrast, macrovesicular steatosis, seen in other conditions, may result from a broader range of mechanisms, including enhanced de novo lipogenesis, impaired VLDL secretion, or reduced β-oxidation (Begriche et al. 2011; Massart et al. 2013). Clinically, patients may exhibit mild to moderate alterations in liver function tests, including elevated bilirubin, reflective of overall hepatic metabolic dysfunction. In contrast to inflammatory phenotypes, liver enzyme elevations (e.g., ALT and AST) may be subtle or delayed in the early stages of toxicity.

Notably, the initial stages of drug-induced lactic acidosis are typically devoid of an inflammatory response. However, as the condition progresses, secondary liver injury can lead to the development of inflammation and potentially more serious liver complications, such as coagulation alterations, hepatic failure or multiorgan dysfunction. The rapid onset and severity of lactic acidosis demand prompt recognition and intervention, as untreated cases can lead to life-threatening outcomes.

#### Cluster 4: Compounds inducing significant hepatic biochemical alterations and mild inflammation

This cluster of compounds is characterized for inducing a moderate inflammatory response in the liver, resulting in steatohepatitis. Clinically, it displays moderate biochemical abnormalities, primarily involving liver enzymes. ALP, ALT and AST levels typically range from 90 to 700 U/L, indicating significant hepatocellular injury. However, bilirubin levels usually remain normal or only mildly elevated, suggesting limited or localized liver dysfunction with preserved bile formation and bilirubin clearance. Compared to cluster 2, this cluster shows a shorter time to onset and slightly higher bilirubin levels. The overall degree of inflammatory activity is typically mild; however, with sustained drug exposure, there is a risk of progression to more advanced liver pathology, including fibrosis and, eventually, cirrhosis.

#### Cluster 5: Steatohepatitis with marked elevations in hepatic enzymes

This phenotype is characterized by a marked elevation in ALT and AST levels (typically > 700 U/L), with only a mild increase in ALP, indicating predominant hepatocellular injury. Inflammation is the primary mechanism driving liver injury in this context. Histologically, the liver typically shows lobular inflammation and ballooning degeneration of hepatocytes, with minimal or no fibrosis during early stages. However, with ongoing exposure, hepatocellular injury may be accompanied by steatosis and sustained inflammation, potentially accelerating progression to fibrosis or cirrhosis. Despite the high transaminase levels indicating hepatocellular damage, overall liver function is often preserved, as suggested by only a moderate increase in serum bilirubin. Alcohol consumption may act as a cofactor, exacerbating drug-induced steatohepatitis in this DIFLD phenotype.

#### Cluster 6: Compounds causing steatohepatitis with associated cholestatic features

This phenotype is characterized by a rapid onset of an inflammatory liver condition, accompanied by greatly elevated bilirubin levels alongside notable increases in ALP, AST, ALT, and GGT. Histological analysis consistently demonstrates the occurrence of cholestasis. A defining feature of this cluster is the presence of bile retention or ductular injury, as indicated by elevated alkaline phosphatase (ALP) and gamma-glutamyl transferase (GGT) levels. These biochemical abnormalities suggest not only hepatocellular injury but also possible biliary epithelial damage or impaired bile flow. Emerging evidence indicates that comorbidities such as diabetes and hypertension may increase susceptibility to drug-induced liver injury within this phenotype cluster.

To further evaluate the biological and clinical relevance of the identified clusters, we retrieved from LiverTox the DILI likelihood scores (A to E) of the drugs of our data set, “A” being the highest and “E” the lowest probability to display hepatotoxicity. For those compounds not classified or scored in LiverTox, estimated scores (in italics) were inferred from the U.S. Food and Drug Administration (FDA) DILIrank.

Our findings (Table 1) reveal a clear and consistent pattern: Cluster 0, used as a control group of non-steatogenic compounds, contains no drugs classified in category A, rather it includes multiple agents with category E or estimated equivalents (e.g., amikacin, diphenhydramine, gentamycin, and salbutamol), all lacking clinical or histological evidence of steatosis. Although omeprazole and tigecycline are listed as B, none of the drugs are associated with steatosis in the LiverTox narrative.

In contrast, Clusters 1 through 6, which represent a gradient of increasing severity and complexity in steatosis phenotypes, consistently include drugs categorized as A or B in LiverTox, with at least one category A drug present in every cluster. This pattern supports the robustness of our phenotypic stratification and reflects the biological plausibility of the identified clusters. Notably, Cluster 3, which features lactic acidosis and mitochondrial toxicity, includes several nucleoside analogues, such as didanosine, stavudine and zidovudine, all known hepatotoxins. Similarly, Clusters 5 and 6, associated with more advanced injury (including steatohepatitis and cholestasis), are enriched with drugs, such as minocycline, nefazodone, valproate, and isoniazid, all of which have a well-documented DILI risk.

Overall, this comparative analysis reinforces the construct validity of our stratification and supports the interpretation of hepatic steatosis as a distinct and clinically relevant phenotype within the broader DILI spectrum.

### Mechanisms implicated in the different DIFL phenotypes and drug clusters

**Fig. 2:**
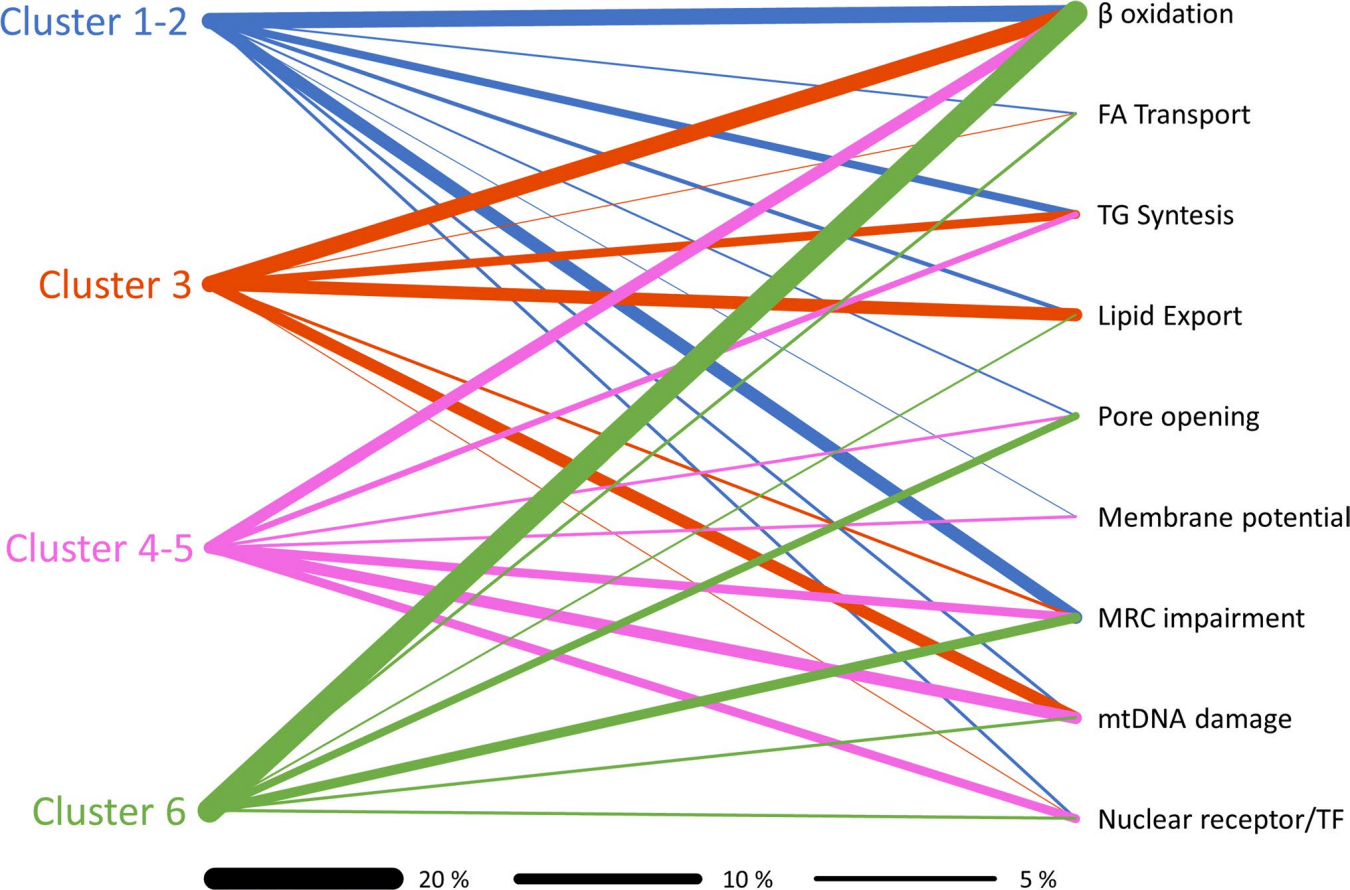
Graphic, semi-quantitative representation of the mechanistic contribution to drug-induced steatosis across clusters. The diagram illustrates the involvement of different toxicity mechanisms in DIFLD for each drug cluster. For each cluster, the total line thickness corresponds to 100% of the mechanistic attribution, while the relative widths depict the proportional contribution of individual mechanisms to DIFLD. Colours indicate the cluster type

To elucidate the ontogeny of disease and its connection to clinical manifestations, we examined the mechanisms of action of drugs associated with the various DIFLD phenotypes. Drawing on our cluster's previously established classification of primary events and molecular mechanisms underlying drug-induced fatty liver injury (López-Pascual et al. 2024), we assigned one or more mechanisms of action to each drug, guided by the number of supporting references in the literature (Fig. 2 and Table S2). Figure 2 visually illustrates the connections between drug clusters and the underlying mechanisms of steatosis, with the thickness of each bar representing the frequency of involvement reported in the literature. At first glance, it is evident that all clusters are linked to multiple mechanisms, though only a few mechanisms dominate within each cluster. This visualization highlights the complex and overlapping molecular pathways involved in steatosis, emphasizing the multifactorial nature of drug-induced liver injury. It demonstrates how different drug clusters contribute through distinct, yet sometimes shared, mechanisms that influence the severity and clinical presentation of liver damage. By clarifying these connections between molecular processes and phenotypes, the analysis aids in identifying potential risk factors and therapeutic targets.

Each cluster of compounds is connected to potential steatotic mechanisms by lines whose thickness is proportional to the percentage of drugs within the cluster associated with each mechanism, based on literature. The total line thickness per cluster represents 100% of the mechanistic attribution for that cluster, illustrating the relative distribution of β-oxidation inhibition, fatty acid (FA) transport alteration, triglyceride (TG) synthesis, impaired lipid export, mitochondrial pore opening, membrane potential disruption, mitochondrial respiratory chain (MRC) impairment, mtDNA (Mitochondrial DNA) depletion, and modulation of nuclear receptors or transcription factors (TF). Colours are related to the cluster type. Thickness of linking lines to the degree of participation of each mechanism. Clusters 1–2 are associated with mild to moderate liver enzyme changes, mainly driven by β-oxidation inhibition and MRC dysfunction. Cluster 3 is characterized by mtDNA depletion and defective lipid export, leading to lactic acidosis without early inflammation. Clusters 4–5 represent inflammatory steatosis with moderate-to-high liver enzyme elevation, where MRC impairment and TF modulation predominate. Cluster 6 shows a mixed profile of steatosis and cholestasis, marked by β-oxidation inhibition, mitochondrial stress.

The involvement of the different mechanisms across the clusters reveal distinct patterns of complexity in the steatosis pathways. *Clusters 1–2* evidence the involvement of diverse mechanisms, notably dominated by β-oxidation impairment and mitochondrial respiratory chain (MRC) dysfunction, without evidence of mitochondrial damage, pointing toward a multifactorial mode of action. *Cluster 3*, in contrast to other clusters, is characterized by disruptions in lipid export and mitochondrial DNA damage, which may be linked to its characteristic phenotype featuring lactic acidosis that results from a metabolic shift toward enhanced glycolysis, driven by impaired ATP production.

*Cluster 4–5* appears more selectively associated with MRC impairment and transcription factor modulation, with limited engagement of other pathways. MRC impairment refers to dysfunction in the mMRC, which is a series of protein complexes embedded in the inner mitochondrial membrane that drive ATP production through oxidative phosphorylation. When there is MRC impairment, these complexes do not function properly, leading to decreased energy production, increased production of reactive oxygen species, and potential cellular damage and subsequent inflammatory response (Urra et al. 2016). Transcription factor modulation involves changes in the activity or expression of transcription factors—proteins that bind to specific DNA sequences to control the rate of gene transcription. Modulation can either enhance or suppress the expression of target genes, thereby influencing various cellular processes. In this context, inflammatory cytokines or oxidative stress can alter the expression of genes involved in lipid metabolism, inflammation, or stress responses, potentially contributing to the disease process (Begriche et al. 2011).

*Cluster 6* is characterized by a dominant contribution from β-oxidation inhibition and a relatively higher involvement of mitochondrial pore opening, and a strong metabolic and mitochondrial stress component. This mechanistic profile may also affect anion and bile acid transporters (Saran and Brouwer 2023), providing a possible explanation for associated cholestatic features.

### Physicochemical properties of drugs across the different clusters

**Fig. 3:**
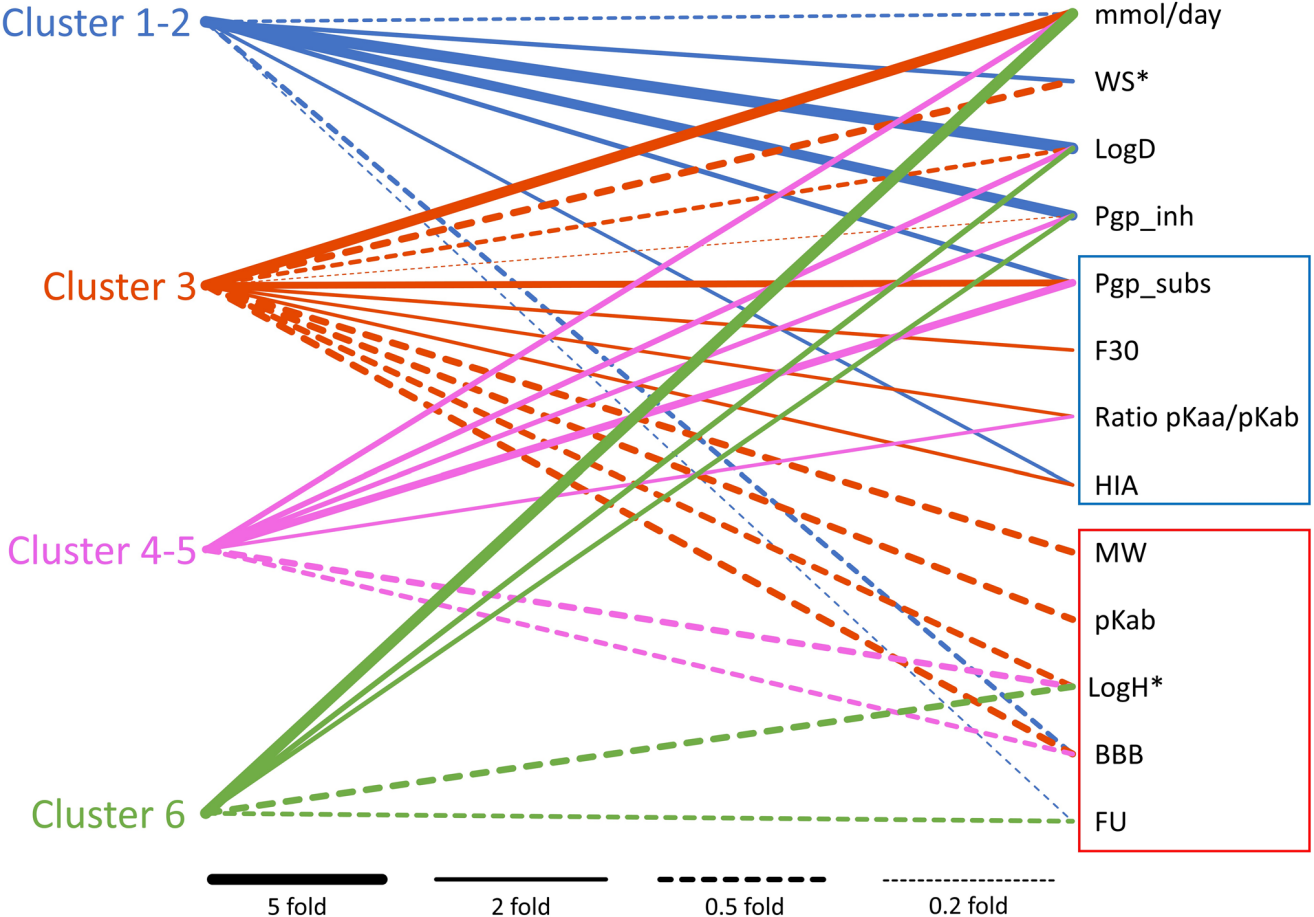
Visualization of the links among drug clusters and the physicochemical properties of drugs. mmol/day Daily dose in millimoles; MP Melting Point; pKab Acid dissociation constant (base); logH* Log hydrophobicity (lipophilicity); BBB Blood-brain barrier penetration; FU Fraction unbound in plasma; WS* Water solubility; logD Distribution coefficient; F30 Oral bioavailability ≥ 30%; ratio pKaa/pKab Ratio of acid/base dissociation; pgp_subs P-glycoprotein substrate status; pgp_inh P-glycoprotein inhibition potential; HIA Human intestinal absorption

Various QSAR models were used to best estimate key physicochemical and ADME properties of the studied drugs, along with their commonly used daily doses, to explore the relationship between chemical characteristics and the different phenotypic clusters. Our analysis revealed notable differences in these properties across the clusters (Fig. 3 and Tables S3, S4). In Fig. 3, we have graphically described the involvement of relevant physicochemical features in the different clusters. Compared to *Cluster 0* (non-steatotic compounds), the other clusters show distinct physicochemical profiles that may relate to their steatogenic potential.

This parallel coordinate plot connects clusters of drug-induced steatosis (left) with daily dose and physicochemical and pharmacokinetic properties (right). Lines represent relationships between steatosis clusters and specific molecular characteristics, summarizing the fold changes in cluster-aggregated mean values relative to Cluster 0 (non-steatotic compounds). Only relationships showing a change greater than ± 50% are displayed—solid lines indicate increased values, while dashed lines indicate decreased values. Line thickness reflects the magnitude of the fold change. Reference lines for fold changes of 5, 2, 0.5 and 0.2 are included for comparison. Variables marked with an asterisk (*) had negative average values; therefore, fold changes are shown in absolute terms. The mean value in LogD for Cluster 3 is negative and opposite in direction to the reference cluster; the reduction is expressed as absolute magnitude. Variables that were exclusively decreased in one or more steatotic clusters are highlighted with a red frame, while those that were exclusively increased are highlighted in blue. This visual distinction helps identify patterns of selective increase or decrease of physicochemical parameters across the clusters. Following parameters were considered: mMol/day-Daily dose in millimoles; MP – Melting Point; pKab – Acid dissociation constant (base); logH* – Log hydrophobicity (lipophilicity); BBB – Blood–brain barrier penetration; FU – Fraction unbound in plasma; WS* – Water solubility; logD – Distribution coefficient; F30 – Oral bioavailability ≥ 30%; ratio pKaa/pKab – Ratio of acid/base dissociation; pgp_subs – P-glycoprotein substrate status; pgp_inh – P-glycoprotein inhibition potential; HIA – Human intestinal absorption. Fold change was calculated using absolute values; the variable showed a 59% decrease in magnitude with a reversal in sign, indicating a negative value in the experimental cluster compared to the control. Clusters 1–2 show mild/moderate steatosis and are associated with low dose, high logD and moderate WS. Cluster 3 is linked to lactic acidosis and displays high dose, low MW, low logD, and high HIA. Clusters 4–5 correspond to inflammatory steatosis with moderate dose, high logD, and low BBB. Cluster 6 combines steatosis and cholestasis, with high logD, low FU, and strong pgp inhibition.

**Clusters 1–2** are characterized by low daily dose exposure (mmol/day), reduced unbound plasma fraction (FU), and increased water solubility (WS). Although these compounds exhibit good aqueous solubility, they also display elevated lipophilicity (logD), suggesting an amphiphilic profile, these compounds display elevated lipophilicity (logD). Their marked interaction with P-glycoprotein (*Pgp*), both as substrates and inhibitors, may influence intracellular accumulation and mitochondrial susceptibility. These properties are consistent with a multifactorial but milder steatogenic profile.

**Cluster 3**, in contrast, presents the highest daily dose and the lowest molecular weight (MW) among all clusters, indicating a predominant role of systemic exposure. Notably, its compounds display markedly reduced lipophilicity (logD), reflecting a hydrophilic profile. Despite this, they are efficiently absorbed (elevated HIA) and exhibit strong interactions as P-gp substrates, which may influence intracellular availability through transporter-mediated distribution. These characteristics suggest that dose intensity, active transport, and mitochondrial susceptibility—not passive accumulation—could be key factors contributing to lipid handling disturbances, such as mtDNA depletion or impaired lipid export.

**Clusters 4–5** exhibit decreased blood–brain barrier permeability (*BBB*), moderately high *logD*, and frequent classification as *Pgp* substrates. These features, combined with intermediate exposure levels (*mmol/day*), may support mechanisms involving membrane interactions and nuclear signalling—especially those tied to MRC impairment and transcription factor modulation.

**Cluster 6** is characterized by high daily dose exposure (mmol/day), elevated lipophilicity (logD), and increased potential to inhibit P-glycoprotein (P-gp), suggesting enhanced hepatic uptake and intracellular retention. Despite the lower unbound plasma fraction (FU), which typically reduces systemic availability, the overall exposure and lipophilicity may favour liver accumulation. These combined features could enhance the local concentration of compounds in hepatocytes, facilitating mitochondrial interaction and contributing to a pro-steatotic effect.

These combinatorial patterns may not manifest as strong univariate correlations when each parameter is considered in isolation, but become evident in cluster-based, multivariable analysis, a key methodological choice in this study. As such, they reflect the nonlinear, multivariate nature of drug-induced steatosis, which we aimed to capture using cluster stratification rather than traditional correlation alone.

## Discussion

### Clinical relevance of the steatotic drug classification

This study provides a comprehensive characterization of DIFLD, integrating clinical phenotypes, underlying mechanisms, and physicochemical profiles of implicated drugs. By exploring the ontogeny of DIFLD, we aimed to understand how these domains interconnect to shape the disease landscape.

Upon large-scale evaluation of scientific literature on DIFLD, we have identified different phenotypes of the disease and drugs associated with those phenotypes. The dendrogram and clustering of drugs, initially suggested by clinician expertise, is sustained by analytical, medical imaging and natural history of the disease. The resulting classification seems a valuable tool to interpret the severity of these adverse reactions posed by pharmaceuticals as well as to anticipate clinical measures and handling of patients. Classifying drugs by their potential to cause fatty liver disease can help physicians anticipate risks, personalize treatment, and improve monitoring, especially for structurally or functionally similar compounds.

The characteristics of the different phenotypes are summarised in Table S5. Thus, a drug with minimal biochemical or inflammatory changes would be classified in **Cluster 1**, where clinicians could anticipate a slow, progressive accumulation of liver fat. Here the role of coexisting metabolic conditions, such as obesity, diabetes or dyslipidemia (Andrade et al. 2019) can significantly amplify the effects of drug-induced liver damage. Monitoring liver function over time would help manage the risk. Understanding the mild yet progressive nature of steatosis associated with these drugs is critical for managing patients with risk factors and optimizing long-term therapeutic strategies.

Drugs associated with mild elevations in ALT and AST, as seen in **Cluster 2**, may indicate a risk for moderate steatosis. While these changes are often subclinical, drugs in this cluster can predispose susceptible individuals, particularly those with metabolic comorbidities like obesity to worsening liver function. Hence, doctors should conduct more frequent liver function tests and metabolic monitoring, to prevent the progression to steatohepatitis or other forms of chronic liver disease if not adequately managed.

Multiple studies demonstrate that patients with metabolic disorders have a higher risk of developing drug-induced liver injury (DILI) compared to those without such conditions. Obesity and nonalcoholic fatty liver disease are particularly important risk factors. Obese individuals or those with NAFLD may experience more severe or more frequent acute liver injury from certain drugs, and some medications can trigger the progression from simple fatty liver to steatohepatitis or worsen necroinflammation and fibrosis (Allard et al. 2019). The underlying mechanisms include mitochondrial dysfunction, increased oxidative stress, impaired fatty acid metabolism, and exacerbation of insulin resistance, all of which can make the liver more susceptible to drug toxicity (Satapathy et al. 2015).

Consistent with expectations, Clusters 0, 1, and 2 are located together in the dendrogram, suggesting similar clinical and histopathological profiles. These clusters are clearly separated from Clusters 3–6, which include drugs associated with severe hepatotoxicity, highlighting their distinct and potentially more aggressive patterns of liver injury.

**Cluster 3** has the drugs associated with the most serious adverse outcomes. They can cause mitochondrial DNA damage, a decrease in mitochondrial number, and a profound reduction in metabolic function, often leading to increased lactic acidemia, a particularly severe complication. Drug-induced steatosis associated with lactic acidosis is a severe and potentially life-threatening condition. This suggests a high risk for rapid-onset lactic acidosis and severe liver injury. In such cases, close monitoring of lactate levels, acid–base status, and liver function is essential, especially in high-risk patients. Cases have reported acute fatty liver with lactic acidosis and hepatic dysfunction progressing inexorably if the offending drug is not promptly discontinued. Effective clinical management should involve vigilant surveillance of these parameters to prevent serious metabolic and hepatic complications and prompt discontinuation of the offending drug (LiverTox 2019a).

Drugs causing moderate increases in ALT, AST, and GGT without dramatic elevations in bilirubin and mild inflammation best fit into **Cluster 4**. Clinicians can expect the slow development of steatohepatitis and monitor liver enzymes regularly to intervene early, potentially discontinuing or modifying the treatment to prevent progression to fibrosis or cirrhosis. Careful monitoring of liver enzymes, especially ALT, AST, ALP, and GGT, is essential in patients receiving these drugs. Early detection of enzyme elevation allows for timely intervention, potentially reducing the risk of progression to more severe liver pathology. In addition, if the drug causes elevations in ALP and GGT, indicating cholestatic or ductular injury, physicians should be cautious, especially in patients prone to cholestasis or with a history of biliary disease. Close monitoring of cholestasis markers would allow early intervention before serious liver dysfunction occurs.

In situations where a drug causes very high ALT and AST elevations, the drug would be placed in **Cluster 5**, indicating a risk for with a pattern of microvesicular steatosis and severe liver injury, particularly in individuals with alcohol use disorder or pre-existing liver conditions. This classification alerts doctors to the potential for rapid liver deterioration in such patients, prompting caution or avoidance of the drug in these high-risk clusters. Regular liver enzyme tests and liver ultrasounds would be crucial during treatment to detect early signs of severe damage or inflammation, enabling timely intervention. Recognizing pre-existing conditions as critical risk factors allows for tailored treatment plans, such as adjusting dosages or selecting alternative therapies to minimize hepatic risks.

Both **Clusters 4 and 5** are characterized by mitochondrial impairment and transcription factor modulation. The progression from simple drug-induced fatty liver disease (DIFLD) to drug-induced steatohepatitis (DISH) involves a multifactorial interplay of drug properties, host metabolic state, and genetic predispositions. Following factors are key in promoting this transition: First a ground basis of Mitochondrial Dysfunction with impaired fatty acid β-oxidation and the electron transport chain, and second, an increase in reactive oxygen species (ROS) production, which triggers oxidative stress, hepatocyte necrosis, and inflammation, hallmarks of steatohepatitis (Chiang and McCullough 2014; Dornas and Schuppan 2020; Li et al. 2022). Excessive ROS formation not only causes lipid peroxidation, generating toxic by-products, such as malondialdehyde (MDA) and 4-hydroxynonenal (HNE), but also the release of pro-inflammatory cytokines (e.g., TNF-α), leading to necroinflammation and fibrosis, as well the activation of hepatic transcription factors, such as SREBP-1c, PPARγ, and PXR, that promote de novo lipogenesis, further fat accumulation and inflammation (Satapathy et al. 2015; Dash et al. 2017; Tang et al. 2022).

Finally, if a new drug causes significant elevations in bilirubin, ALP, ALT and a AST, it would fall under Cluster 6—indicating cholestasis with an inflammatory component. While drug-induced liver steatosis and cholestasis are distinct conditions with different mechanisms and clinical features, they can occur together. This overlap is seen in some cases of drug-induced liver injury (DILI), where a single drug or its metabolites simultaneously disrupt lipid metabolism and bile flow (Satapathy et al. 2015; García-Cortés et al. 2023). For instance, some drugs impair mitochondrial function, leading to steatosis, while also inhibiting bile acid transporters like the bile salt export pump (BSEP), resulting in cholestasis. Valproic acid (VPA) exemplifies a drug that induces both hepatic steatosis and cholestasis through dual mechanisms involving mitochondrial dysfunction and inhibition of bile acid transporters. VPA disrupts mitochondrial β-oxidation of fatty acids, leading to lipid accumulation within hepatocytes and subsequent steatosis (Fu et al. 2019; López-Pascual et al. 2024; Kadam et al. 2025). In cases of acute microvesicular steatosis, significant cholestasis is rare due to the rapid progression of liver damage. However, with chronic exposure or in susceptible individuals, steatosis and cholestasis may coexist, producing a mixed pattern of liver injury characterized by elevations in both aminotransferases and cholestatic markers, such as ALP and bilirubin. Frequent monitoring of bilirubin and ALP levels would be essential, and clinicians could act quickly if signs of cholestasis appear, potentially discontinuing the drug or switching to a safer alternative to prevent further liver damage. This approach helps mitigate the risk of prolonged cholestasis, which could otherwise lead to more severe liver dysfunction (Satapathy et al. 2015).

When compared with the DILI likelihood score (Table 1), there are few apparent disparities. Lomitapide (Cluster 1) is scored as C in LiverTox, although the same database notes that “Lomitapide is associated with mild, asymptomatic and self-limited serum aminotransferase elevations during therapy that are usually accompanied by an increase in hepatic fat”. Hence this description aligns with our clinical Cluster 1 phenotype. Capecitabine (Cluster 4) is listed as E*, indicating a suspicion without conclusive evidence of DILI. Nevertheless, its active metabolite 5-fluorouracil (5-FU), also present in Cluster 4, is a clear category A hepatotoxin with known features of hepatic steatosis. The clustering of both drugs is thus fully justified.

Finally, raloxifene (Cluster 2) was not listed in LiverTox and is classified as “No DILI concern” in the FDA DILIrank database. However, clinical evidences supported its potential to aggravate pre-existing hepatic steatosis, rather displaying a general hepatotoxic risk per se.

One has to be aware that while the LiverTox system reflects the overall likelihood of a drug being associated with hepatotoxicity, regardless of the type and nature of the injury, our clustering is based specifically on clinical and histopathological data associated with drug-induced hepatic steatosis. Moreover, some steatosis-associated DILI could present with mild symptoms or asymptomatic.

In summary, there is a great deal of coincidence among our phenotypic clustering and that of LiverTox and they converge meaningfully: steatotic clusters are largely composed of drugs with established or suspected hepatotoxicity, while the control group is clearly distinct. This convergence supports the construct validity of our clustering approach and reinforces the relevance of steatosis as a distinct and clinically meaningful phenotype within DILI. This proactive approach supports early detection of liver toxicity, reducing the risk of progression to more serious conditions and ensuring safer therapeutic outcomes. The classification reflects various toxicity mechanisms and chemical properties, ranging from mild fat accumulation in the liver to severe metabolic disruptions like lactic acidosis. By applying this classification system, healthcare providers could better anticipate drug-induced liver risks, personalize patient care, and implement early interventions, ultimately improving patient safety and therapeutic outcomes.

### Mechanistic pathways and pro-steatotic potential of drugs

By examining the mechanisms of action of drugs associated with various DIFLD phenotypes, we identified a range of clinical presentations, from asymptomatic steatosis to steatohepatitis and cirrhosis, and sought to link these to previously established DIFLD mechanisms to better understand the development (ontogeny) and clinical expression of drug-induced fatty liver (DIFL). Using our earlier classification of primary events and molecular mechanisms in DIFLD (López-Pascual et al. 2024), we assigned one or more mechanisms to each drug based on the number of supporting literature references.

DIFLD arises from a complex interplay of molecular mechanisms, primarily involving disruptions in mitochondrial function, lipid metabolism, and gene regulation. The pro-steatotic potential of a drug depends on its ability to interfere with these pathways, often in a dose- and patient-dependent manner (Di Pasqua et al. 2022). Figure 2 visually maps the relationship between drug clusters and steatosis mechanisms and reflects the frequency of each mechanism's involvement, and provides a semi-quantitative visualization of these relationships. As observed, all clusters are linked to multiple mechanisms and the distribution of implicated mechanisms across drug clusters revealed distinct patterns of mechanistic complexity. Collectively, these lines show that most clusters are influenced by multiple overlapping mechanisms.

Clusters 1–2 demonstrated the involvement of multiple pathways, predominantly β-oxidation impairment and MRC dysfunction. Interestingly, despite the mitochondrial involvement, there was no evidence of direct mitochondrial damage, suggesting a multifactorial mode of action. Clusters 3–5 exhibit more complex profiles. Cluster 3 exhibited a distinct mitochondrial signature, marked by mtDNA depletion, reduced mitochondrial content, and a shift toward glycolysis—all likely stemming from impaired ATP production via oxidative phosphorylation. In addition, there was evidence of disrupted lipid export, indicating a complex, mitochondria-driven phenotype. Clusters 4–5 were more selectively linked to MRC impairment and transcription factor modulation, with minimal engagement of other pathways. MRC impairment refers to dysfunction in the mitochondrial electron transport chain, leading to reduced ATP synthesis, elevated reactive oxygen species (ROS), and downstream cellular damage and inflammation. Transcription factor modulation involves altered activity of nuclear regulators controlling genes involved in lipid metabolism, stress responses, and inflammation. This modulation may be driven by inflammatory cytokines or oxidative stress, further contributing to steatosis. Cluster 6 was characterized by β-oxidation inhibition, increased mitochondrial permeability transition pore (MPTP) opening, and broader signs of metabolic and mitochondrial stress. These alterations are strongly associated with impaired fatty acid metabolism and mitochondrial dysfunction, which are known contributors to hepatic lipid accumulation and the onset of steatosis (Begriche et al. 2011). In parallel, the same mitochondrial disruptions can impair the function of bile acid and anion transporters, such as the bile salt export pump (BSEP), leading to intracellular bile acid accumulation. This mechanism likely underlies the cholestatic features observed in this cluster, linking mitochondrial dysfunction to impaired bile flow and hepatocellular injury (Saran and Brouwer 2023).

The phenotypic clustering revealed distinct subclusters or patterns associated with specific drug classes or mechanisms. This heterogeneity may reflect differences in patient susceptibility, drug exposure duration, or coexisting metabolic conditions. Our findings reinforce the mechanistic diversity underlying DIFLD, with contributions from mitochondrial dysfunction, oxidative stress, impaired lipid export, and autophagy inhibition. Importantly, certain drugs were associated with more than one mechanistic pathway, supporting the concept of multifactorial toxicity.

This visualization underscores the overlapping yet distinct molecular pathways underlying steatosis, highlighting the multifactorial nature of drug-induced liver injury. It supports the notion that drug clusters contribute to steatosis via distinct, sometimes overlapping, mechanisms that shape the severity and phenotype of liver injury. Overall, the analysis aids in identifying potential risk factors and therapeutic targets by clarifying the link between molecular mechanisms and clinical phenotypes.

These mechanistic insights align with the known pathophysiology of non-alcoholic fatty liver disease (NAFLD), suggesting that DIFLD may serve as a model to study specific disruptions in hepatic lipid homeostasis. Recognizing these phenotypic variants is crucial for improving diagnosis and tailoring monitoring strategies, especially in polypharmacy settings.

### Link between physicochemical properties and pro-steatotic potential

The physicochemical properties of drugs play a critical role in determining their pro-steatotic potential. Consistent relationships have emerged when comparing mechanistic clusters with their chemical profiles. The analysis of these properties, adjusted for typical daily doses (in millimoles), revealed marked differences between clusters—particularly between Clusters 1–2 and Cluster 3—when compared to non-steatotic compounds (Cluster 0).

Clusters 1–2, associated with milder, multifactorial mechanisms, are characterized by low daily doses (mmol/day), moderate plasma protein binding (higher fraction unbound, FU), and elevated water solubility (WS). These compounds also exhibit lower blood–brain barrier permeability (BBB) and moderate P-glycoprotein interaction (as both substrates and inhibitors). Their overall lipophilic profile (high logD) may promote intracellular access, possibly favouring mitochondrial accumulation and interference with β-oxidation (Begriche et al. 2011).

In contrast, Cluster 3—strongly associated with mtDNA depletion and impaired lipid export—displays the highest daily exposure (mmol/day) among all clusters, combined with moderate lipophilicity (logD), relatively low water solubility (WS), and increased interaction as a P-glycoprotein substrate (Pgp subs). These characteristics suggest that systemic exposure and transporter-mediated hepatic uptake, rather than passive membrane diffusion, are likely key contributors to its mitochondrial toxicity. This is consistent with the “rule-of-two”, which links drugs with high daily doses and lipophilicity to a greater risk of drug-induced liver injury (DILI) (Lewis 2015). In addition, studies suggest hydrophilic compounds may reach mitochondria via active transport mechanisms or diffuse into mitochondria, contributing to functional impairment and supporting a broader view of mitochondrial vulnerability (Lai et al. 2004; Mukhopadhyay and Weiner 2007).

Clusters 4–5, primarily associated with MRC impairment and transcription factor modulation, exhibit moderate exposure, high lipophilicity, and reduced BBB permeability, indicating a profile driven more by membrane and nuclear signalling interactions. Lipophilic drugs are known to partition into membranes and accumulate in lipid droplets, disrupting mitochondrial function and fatty acid oxidation (Fromenty and Pessayre 1995; Lettéron et al. 2003; Pessayre et al. 2012).

Similarly, Cluster 6—linked to β-oxidation inhibition and mitochondrial permeability transition pore opening—shows low plasma availability (low FU), high lipophilicity and strong P-gp inhibition, suggesting increased hepatic retention and a predisposition to mitochondrial stress (Smith et al. 2003; Jabeen et al. 2012; Contino et al. 2013).

Notably, drugs linked to different DILI phenotypes tend to share specific physicochemical traits, particularly lipophilicity, moderate-to-high molecular weight, and defined pKa distributions. These properties may enhance hepatic accumulation, especially in lipid-rich environments, thereby increasing the potential for steatogenic damage. Such patterns of lipophilic accumulation and mitochondrial disruption are well-documented in experimental hepatotoxicity models (Lettéron et al. 2003; Fromenty and Pessayre 1995).

Our approach to consider physicochemical parameters is deeply rooted in toxicological principles and builds upon well-established frameworks in the field of Predictive Toxicology (Yukawa and Naven 2020): as numerous toxicological studies have demonstrated that physicochemical properties such as lipophilicity, water solubility, plasma protein binding, and transporter interaction play determinant roles in hepatic drug disposition, intracellular accumulation, and mitochondrial toxicity, and hence key drivers of DIFL we do not suggest that physicochemical profiling replaces mechanistic studies. Rather, it serves as a complementary tool, particularly in early drug development, by flagging compounds with physicochemical traits associated with known toxicological outcomes. The value of physicochemical profiling lies in its predictive capacity, especially when empirical in vivo or in vitro data are limited. By linking distinct chemical signatures to steatosis clusters, we contribute to the ongoing efforts in predictive toxicology to identify early warning signs of hepatotoxicity, which is of critical importance in drug development pipelines.

These findings carry important implications for early drug development, where physicochemical “flags” could be incorporated into predictive models of hepatotoxicity. For instance, machine learning tools such as pDILI_v1 integrate descriptors such as SLogP and molecular fingerprints to estimate DILI risk, emphasizing the relevance of lipophilicity and structural alerts (Amin et al. 2025). Understanding these connections may help in predicting the risk of steatosis based on drug properties, potentially guiding safer drug design.

### Ontogeny of DIFLD

The concept of ontogeny in the context of drug-induced fatty liver disease (DIFLD) refers to the dynamic and multifactorial progression of hepatic steatosis triggered by pharmaceutical agents. Our integrative approach underscores the ontogeny of DIFLD as a complex interplay between drug properties, mechanistic pathways, and host responses. This framework provides a path forward for categorizing DILI cases with steatogenic features, moving beyond traditional binary classifications. Our analysis supports a framework in which DIFLD emerges from the intersection of three interconnected domains: the physicochemical properties of drugs, the biological mechanisms they perturb, and the clinical phenotypes that result. This system-level perspective moves beyond a simplistic one-drug–one-effect paradigm and instead embraces the idea that DIFLD represents a spectrum of injury shaped by both compound-specific factors and host susceptibilities.

First, the physicochemical characteristics of drugs—particularly their lipophilicity, molecular weight, and ability to undergo hepatic metabolism—appear to likely influence their propensity to accumulate in hepatocytes and interact with intracellular lipid handling pathways. Lipophilic compounds, for example, are more likely to localize in hepatic lipid droplets and membranes, which would be in agreement with their potentially disrupting mitochondrial function, β-oxidation, or vesicular trafficking. Moreover, our clustering results also highlight other alternative profiles: certain hydrophilic, low-molecular-weight compounds (e.g., Cluster 3) can exert potent mitochondrial toxicity as well, through mechanisms, such as enzyme inhibition or mtDNA depletion, without requiring membrane accumulation. This indicates that steatogenic stress may arise from both membrane-associated and direct molecular interactions, particularly under conditions of prolonged exposure or metabolic vulnerability.

Second, the mechanisms by which these compounds disrupt hepatic lipid homeostasis are varied but often converge on a limited set of biological pathways, including mitochondrial dysfunction (mitochondrial respiratory chain, membrane potential, mtDNA damage, pore opening) nuclear receptors and transcription factor modulation and impaired fatty acid or lipid synthesis, oxidation or transport. Some drugs perturb a single dominant pathway, while others may act via multiple mechanisms, amplifying the risk of more severe or persistent liver injury. This mechanistic heterogeneity underscores the importance of pathway-level annotations in understanding DIFLD risk of drugs.

Third, these mechanistic perturbations give rise to distinct clinical phenotypes, ranging from isolated steatosis to steatohepatitis, fibrosis, or even cirrhosis. Our analysis revealed that certain mechanistic signatures (e.g., mitochondrial inhibition and mtDNA damage) were more commonly associated with the more severe phenotypes, suggesting a trajectory of disease progression that parallels non-alcoholic fatty liver disease (NAFLD) but is initiated by pharmacological triggers. Moreover, the phenotype may be influenced by host factors, such as metabolic comorbidities and concurrent drug exposures, adding an additional layer of complexity to the ontogeny model.

By integrating these domains, we propose that the ontogeny of DIFLD can be conceptualized as a multi-stage process initiated by physicochemical entry points, modulated by mechanistic disruptions, and expressed as a spectrum of hepatic phenotypes. This framework has both scientific and translational value: it facilitates hypothesis generation regarding the hepatotoxic potential of new drugs, supports mechanistic classification of DILI cases, and may guide the development of predictive models for identifying high-risk compounds and patients.

This study is limited by reliance on literature-derived mechanisms or accuracy of clinical reports. Future work should include experimental validation of proposed mechanisms, incorporation of genetic and metabolic risk factors, and prospective studies to evaluate causality. Incorporating systems biology and AI-based modelling may also help predict DIFLD risk early in the drug development process.These data are publicly available in the BioStudies repository under accession number S-ONTX76.

## Supplementary Information

Below is the link to the electronic supplementary material.

Supplementary file1 (DOCX 57 KB)
