# Supplementary material for: Ontogeny of drug-induced fatty liver disease (DIFLD): from key initiating events to disease phenotypes
Source: Arch Toxicol. 2025 Sep 16;99(12):5075–91. doi: 10.1007/s00204-025-04178-x (PMC12534315; doi:10.1007/s00204-025-04178-x)
Supplement: Supplementary file 1 — Supplementary file1 (DOCX 57 KB) [file 204_2025_4178_MOESM1_ESM.docx]

**Supplementary Material**

**Table S1. Aggregated clinical features from case reports used for drug clustering**

|  | **Previous condition** | | | **Dose** | | **Clinical manifestations** | | | | | | | |
| --- | --- | --- | --- | --- | --- | --- | --- | --- | --- | --- | --- | --- | --- |
| **CLUSTER** | **Alcohol  (%)** | **Obesity  (%)** | **Diabetes  (%)** | **Dose (mg/day)** | **Total dose (mg)** | **Time to Onset (days)** | **ALP  (UI/L)** | **ALT  (UI/L)** | **AST  (UI/L)** | **GGT  (UI/L)** | **Total Bilirubin (mg/dL)** | **Lactic Acidosis (%)** | **Coagulation alteration (%)** |
| **Cluster 1** | 16 | 13 | 36 | 14 | 11402 | 131 | 143 | 70 | 71 | 82 | 1 | 0 | 70 |
| **Cluster 2** | 6 | 0 | 21 | 133 | 95420 | 348 | 601 | 333 | 320 | 408 | 1 | 0 | 31 |
| **Cluster 3** | 4 | 37 | 2 | 1016 | 58743 | 95 | 384 | 255 | 273 | 373 | 8 | 79 | 89 |
| **Cluster 4** | 13 | 13 | 50 | 817 | 193274 | 49 | 200 | 274 | 364 | 773 | 5 | 0 | 100 |
| **Cluster 5** | 67 | 25 | 0 | 375 | 25500 | 130 | 216 | 1893 | 1326 | 3362 | 6 | 17 | 33 |
| **Cluster 6** | 3 | 4 | 33 | 687 | 62490 | 129 | 463 | 646 | 914 | 786 | 15 | 4 | 72 |

|  | **Histopathological Manifestation** | | | | | | | | | | **MRI/US/Histopathology** | | | **Outcome** | | |
| --- | --- | --- | --- | --- | --- | --- | --- | --- | --- | --- | --- | --- | --- | --- | --- | --- |
| **CLUSTER** | **Micro- steatosis  (%)** | **Macro- steatosis  (%)** | **Micro & Macro steatosis  (%)** | **Cellular  degeneration  (%)** | **Ballooning (%)** | **Mallory bodies  (%)** | **Necrosis  (%)** | **Inflammation (%)** | **Fibrosis OR cirrhosis  (%)** | **Cholestasis (%)** | **Ascites  (%)** | **Hepato- megaly  (%)** | **Spleno- megaly  (%)** | **Fatal  (%)** | **Chronic (%)** | **Recover (%)** |
| **Cluster 1** | 0 | 83 | 17 | 0 | 23 | 20 | 47 | 67 | 85 | 8 | 15 | 21 | 17 | 15 | 44 | 41 |
| **Cluster 2** | 37 | 44 | 19 | 27 | 42 | 36 | 17 | 67 | 59 | 2 | 16 | 26 | 7 | 24 | 3 | 73 |
| **Cluster 3** | 43 | 25 | 32 | 1 | 4 | 4 | 24 | 26 | 15 | 65 | 0 | 25 | 3 | 63 | 5 | 31 |
| **Cluster 4** | 19 | 75 | 6 | 3 | 6 | 12 | 26 | 66 | 57 | 3 | 6 | 0 | 3 | 19 | 35 | 46 |
| **Cluster 5** | 33 | 33 | 33 | 0 | 33 | 0 | 50 | 67 | 17 | 0 | 0 | 0 | 0 | 23 | 17 | 60 |
| **Cluster 6** | 54 | 26 | 19 | 10 | 30 | 8 | 63 | 67 | 23 | 50 | 21 | 24 | 7 | 32 | 9 | 59 |

Distinct clinical features associated with DIFLD events were extracted from the literature and compiled into a structured table. The values shown represent the average computed for each cluster from the medians of clinical parameters reported in case studies, aggregated by drug. A yellow-to-green color gradient was used to represent the absolute values in each column, with green denoting the highest values and yellow the lowest. The retrieved clinical parameters were subsequently analyzed, and tentative, arbitrary cutoffs—based on clinical judgment rather than standardized thresholds—were applied to define boundaries for potential clusters. These clusters served as the foundation for constructing a dendrogram to visualize relationships among the clusters. Cluster 1 includes drugs associated with mild steatosis and near-normal liver parameters. Cluster 2 comprises drugs linked to moderate steatosis and mild liver enzyme elevations. Cluster 3 includes drugs associated with mitochondrial dysfunction and lactic acidosis. Cluster 4 groups drugs that cause steatohepatitis with moderate increases in liver enzymes. Cluster 5 consists of drugs associated with steatohepatitis and marked hepatocellular injury. Cluster 6 includes drugs inducing steatohepatitis with cholestatic features. **ALP**, alkaline phosphatase; **ALT**, alanine aminotransferase; **AST**, aspartate aminotransferase; **GGT**, gamma-glutamyl transferase; **MRI**, magnetic resonance imaging; **US**, ultrasounds.

**Table S2 Mechanistic contribution of individual compounds to drug-induced hepatic steatosis across defined clusters.**

|  | **COMPOUND** | **β oxidation** | **FA Transpor** | **TG Syntesis** | **Lipid Export** | **Pore opening** | **Membrane potential** | **MRC impairment** | **mtDNA depletion** | **Nuclear receptor/TF** |
| --- | --- | --- | --- | --- | --- | --- | --- | --- | --- | --- |
| Cluster 1-2 | Amiodarone | 9 | 2 | 3 | 4 | 3 | 2 | 7 | 0 | 0 |
|  | Methotrexate | 4 | 0 | 1 | 0 | 0 | 0 | 4 | 0 | 2 |
|  | Tamoxifen | 7 | 1 | 6 | 2 | 0 | 0 | 5 | 4 | 2 |
| Cluster 3 | Didanosine | 4 | 0 | 2 | 3 | 0 | 0 | 0 | 4 | 0 |
|  | Stavudine | 5 | 0 | 2 | 4 | 0 | 0 | 0 | 4 | 0 |
|  | Tetracycline | 5 | 1 | 3 | 4 | 0 | 0 | 2 | 0 | 1 |
|  | Zidovudine | 4 | 0 | 2 | 3 | 0 | 0 | 1 | 5 | 0 |
| Cluster 4-5 | Chloroform | 0 | 0 | 0 | 0 | 0 | 0 | 1 | 0 | 0 |
|  | Fluorouracil | 4 | 0 | 0 | 0 | 0 | 0 | 0 | 0 | 0 |
|  | Nefazodone | 0 | 0 | 0 | 0 | 0 | 1 | 1 | 0 | 0 |
|  | Troglitazone | 0 | 0 | 2 | 0 | 1 | 0 | 1 | 4 | 3 |
| Cluster 6 | Doxycycline | 2 | 0 | 0 | 0 | 0 | 0 | 1 | 0 | 1 |
|  | Irinotecan | 3 | 0 | 0 | 0 | 0 | 0 | 2 | 2 | 0 |
|  | Valproic | 8 | 2 | 0 | 1 | 4 | 0 | 2 | 0 | 1 |

The table presents the number of associations reported in the scientific literature between each compound—clustered by phenotype—and nine mechanistic pathways involved in steatosis. These associations were compiled from scientific sources, with López-Pascual et al. (2024) serving as the primary reference. Data for chloroform and nefazodone were integrated from additional sources (Dykens et al. 2008; Silva et al. 2016; Minor et al. 2018): inhibition of β-oxidation, altered fatty acid transport (FA Transport), triglyceride synthesis (TG Synthesis), impaired lipid export, mitochondrial pore opening, membrane potential disruption, mitochondrial respiratory chain (MRC) impairment, mitochondrial DNA (mtDNA) depletion, and modulation of nuclear receptors or transcription factors (Nuclear receptor/TF). Clusters 1–2 involve drugs causing mild to moderate hepatic alterations, predominantly linked to inhibition of β-oxidation and dysfunction of the MRC. Cluster 3 includes agents that compromise mtDNA integrity and lipid export capacity, often associated with lactic acidosis in the absence of significant inflammation. Clusters 4–5 encompass compounds with inflammatory steatosis, frequently associated with elevated transaminases and driven by mitochondrial impairment and TF-mediated pathways. Cluster 6 displays a mixed cholestatic–steatotic profile, with combined effects on β-oxidation, mitochondrial integrity.

**Table S3 Physicochemical and pharmacokinetic parameters for the compounds included in the study**

| **Compound_**  **Name** | **CLUSTER** | **Compound abbreviation** | **CHEMBL ID** | **mmol per day** | **moleular weight (g/mol)** | **pKa-a** | **pKa-b** | **BP  [ºC]** | **MP  [ºC]** | **WS  [logM]** | **logD** | **logH  [log atm-m3/mole]** | **logVP  [log 1/(mmHg)]** | **Caco2  [log cm/s]** | **logKp  [log cm/h]** | **FU** | **F30**  **[class]** | **BBB [class]** | **pgp_inh [class]** | **pgp_subs [class]** | **HIA**  **[class]** |
| --- | --- | --- | --- | --- | --- | --- | --- | --- | --- | --- | --- | --- | --- | --- | --- | --- | --- | --- | --- | --- | --- |
| **Amikacin** | Cluster 0 | AKC | CHEMBL177 | 2.39 | 585.29 | 5.08 | 7.76 | 420.80 | 203.50 | -0.50 | -2.20 | -41.57 | -5.91 | -6.31 | -5.12 | 0.90 | 0 | 1 | 0 | 0 | 0 |
| **Diphenhydramine** | Cluster 0 | DIP | CHEMBL657 | 1.18 | 255.16 | 0.00 | 8.78 | 355.00 | 165.70 | -1.92 | 1.34 | -7.01 | -3.61 | -4.27 | -2.82 | 0.30 | 1 | 1 | 0 | 0 | 1 |
| **Gentamycin** | Cluster 0 | GEN | CHEMBL329592 | 0.73 | 477.32 | 6.79 | 8.39 | 233.10 | 105.00 | -2.66 | -2.10 | -34.97 | -4.88 | -5.72 | -4.69 | 0.96 | 0 | 0 | 0 | 0 | 0 |
| **Levothyroxinie** | Cluster 0 | LEV | CHEMBL1624 | 0.26 | 776.69 | 5.85 | 1.45 | 344.53 | 235.50 | -4.93 | 1.50 | -18.10 | -5.11 | -5.29 | -2.98 | 0.59 | 1 | 1 | 0 | 0 | 1 |
| **Loperamide** | Cluster 0 | LOP | CHEMBL841 | 0.034 | 476.22 | 0.00 | 7.66 | 298.00 | 228.00 | -4.64 | 3.40 | -7.26 | -7.71 | -4.97 | -3.10 | 0.05 | 1 | 1 | 1 | 1 | 1 |
| **Omeprazole** | Cluster 0 | OME | CHEMBL1503 | 0.12 | 345.11 | 12.08 | 4.44 | 276.00 | 156.00 | -3.82 | 2.20 | -9.44 | -6.32 | -4.71 | -2.75 | 0.07 | 1 | 1 | 0 | 0 | 1 |
| **Salbutamol** | Cluster 0 | SAL | CHEMBL714 | 0.04 | 239.15 | 10.12 | 8.96 | 433.00 | 153.83 | -1.23 | 0.39 | -15.19 | -4.68 | -4.97 | -3.56 | 0.84 | 1 | 0 | 0 | 0 | 1 |
| **Tigecycline** | Cluster 0 | TIG | CHEMBL376140 | 0.17 | 585.28 | 6.65 | 8.70 | 268.23 | 222.70 | -2.10 | 0.17 | -28.53 | -7.55 | -5.95 | -4.52 | 0.27 | 0 | 0 | 0 | 0 | 0 |
| **Lomitapide** | Cluster 1 | LOM | CHEMBL354541 | 0.03 | 693.28 | 9.90 | 7.92 | 371.02 | 181.00 | -6.80 | 4.40 | -16.19 | -5.28 | -4.95 | -4.06 | 0.02 | 1 | 0 | 1 | 0 | 1 |
| **Methotrexate** | Cluster 1 | MET | CHEMBL34259 | 0.005 | 454.17 | 1.95 | 3.82 | 386.42 | 195.00 | -3.43 | -2.73 | -30.81 | -6.97 | -5.90 | -4.01 | 0.47 | 0 | 0 | 0 | 0 | 1 |
| **Tamoxifen** | Cluster 1 | TAM | CHEMBL83 | 0.05 | 371.22 | 10.62 | 9.77 | 442.00 | 97.13 | -5.56 | 4.20 | -5.57 | -5.61 | -4.55 | -3.01 | 0.01 | 0 | 1 | 1 | 0 | 1 |
| **Amiodarone** | Cluster 2 | AMI | CHEMBL633 | 0.38 | 645.02 | 12.42 | 5.57 | 456.35 | 111.00 | -4.06 | 6.10 | -6.85 | -7.96 | -4.84 | -2.79 | 0.01 | 1 | 0 | 1 | 1 | 1 |
| **Atorvastatin** | Cluster 2 | ATO | CHEMBL1487 | 0.014 | 558.25 | 8.96 | 3.35 | 316.25 | 159.95 | -7.77 | 1.70 | -22.62 | -5.32 | -5.31 | -3.30 | 0.02 | 0 | 0 | 0 | 1 | 1 |
| **Perhexiline** | Cluster 2 | PER | CHEMBL75880 | 0.79 | 277.28 | 0.00 | 11.11 | 342.00 | 81.00 | -5.14 | 4.20 | -4.35 | -5.17 | -5.01 | -1.56 | 0.04 | 1 | 1 | 1 | 0 | 1 |
| **Raloxifene** | Cluster 2 | RAL | CHEMBL81 | 0.13 | 473.17 | 10.81 | 6.17 | 352.00 | 145.00 | -5.97 | 4.30 | -20.46 | -8.60 | -5.72 | -3.36 | 0.01 | 0 | 0 | 0 | 0 | 1 |
| **Didanosine** | Cluster 3 | DID | CHEMBL1460 | 2.75 | 235.08 | 9.41 | 3.78 | 333.00 | 161.50 | -1.11 | -0.34 | -8.38 | -6.75 | -5.72 | -3.25 | 0.90 | 1 | 0 | 0 | 0 | 1 |
| **Linezolid** | Cluster 3 | LIN | CHEMBL126 | 3.56 | 337.14 | 8.94 | 5.02 | 300.00 | 182.00 | -2.37 | 0.59 | -8.24 | -6.44 | -4.68 | -3.72 | 0.67 | 1 | 1 | 0 | 1 | 1 |
| **Stavudine** | Cluster 3 | STA | CHEMBL991 | 0.36 | 223.07 | 8.92 | 3.38 | 283.00 | 159.50 | -1.15 | -0.61 | -8.62 | -5.08 | -5.35 | -3.37 | 0.63 | 1 | 1 | 0 | 0 | 1 |
| **Tetracycline** | Cluster 3 | TET | CHEMBL1440 | 5.63 | 444.15 | 5.15 | 6.68 | 295.96 | 165.00 | -3.28 | -0.61 | -23.33 | -4.09 | -6.42 | -4.92 | 0.33 | 1 | 0 | 0 | 1 | 1 |
| **Zidovudine** | Cluster 3 | ZID | CHEMBL129 | 2.25 | 266.09 | 7.83 | 3.70 | 242.62 | 110.75 | -1.12 | -0.25 | -9.51 | -7.69 | -5.06 | -3.29 | 0.88 | 1 | 0 | 0 | 0 | 1 |
| **5-Fluorouracil** | Cluster 4 | FLU | CHEMBL185 |  | 128.00 | 6.52 | 1.39 | 196.00 | 284.82 | -1.07 | -0.71 | -7.76 | -1.34 | -5.30 | -3.92 | 0.70 | 0 | 0 | 0 | 0 | 0 |
| **Capecitabine** | Cluster 4 | CAP | CHEMBL1773 | 5.57 | 359.15 | 1.19 | 5.62 | 735.00 | 115.50 | -1.04 | 0.35 | -18.53 | -7.09 | -5.38 | -3.53 | 0.69 | 1 | 0 | 0 | 0 | 1 |
| **Prednisolone** | Cluster 4 | PRE | CHEMBL131 | 0.14 | 360.19 | 8.10 | 3.19 | 331.42 | 240.00 | -3.21 | 1.30 | -7.57 | -7.23 | -4.72 | -4.35 | 0.22 | 1 | 0 | 0 | 0 | 1 |
| **Troglitazone** | Cluster 4 | TRO | CHEMBL408 | 0.91 | 441.16 | 10.35 | 4.07 | 447.00 | 208.00 | -4.64 | 3.30 | -8.56 | -6.62 | -5.33 | -3.05 | 0.01 | 1 | 0 | 1 | 1 | 1 |
| **Chloroform** | Cluster 5 | CHL | CHEMBL44618 |  | 117.91 | 12.29 | 5.99 | 59.55 | -63.60 | -1.18 | 1.88 | 0.00 | -2.29 | -4.38 | -1.80 | 0.57 | 1 | 0 | 0 | 0 | 1 |
| **Minocycline** | Cluster 5 | MIN | CHEMBL1434 | 0.55 | 457.18 | 6.61 | 7.83 | 285.91 | 199.80 | -0.94 | -0.21 | -23.82 | -9.43 | -6.10 | -4.47 | 0.28 | 0 | 1 | 0 | 1 | 1 |
| **Nefazodone** | Cluster 5 | NEF | CHEMBL623 | 1.07 | 469.22 | 9.83 | 7.10 | 304.80 | 82.90 | -5.33 | 4.83 | -16.82 | -8.25 | -5.17 | -3.15 | 0.02 | 0 | 1 | 1 | 1 | 1 |
| **Arsenic trioxide** | Cluster 6 | ARA | CHEMBL5483015 |  | 197.83 | 5.00 | 4.31 | 225.00 | 80.00 | -0.74 | 0.71 | 0.00 | 1.06 | -4.53 | -4.35 | 0.00 | 0 | 0 | 0 | 0 | 0 |
| **Carbamazepine** | Cluster 6 | CAR | CHEMBL108 | 1.69 | 236.09 | 10.21 | 4.85 | 281.00 | 191.60 | -3.32 | 2.90 | -9.23 | -7.38 | -4.41 | -2.12 | 0.18 | 1 | 1 | 0 | 0 | 1 |
| **Doxycycline** | Cluster 6 | DOX | CHEMBL1433 | 0.45 | 444.15 | 6.63 | 6.53 | 289.93 | 201.00 | -2.85 | -0.57 | -23.33 | -4.10 | -5.63 | -4.92 | 0.34 | 1 | 0 | 0 | 0 | 1 |
| **Enalapril** | Cluster 6 | ENA | CHEMBL578 | 0.04 | 376.20 | 7.23 | 5.32 | 396.00 | 143.75 | -1.36 | -1.20 | -15.48 | -7.49 | -5.67 | -3.94 | 0.45 | 0 | 0 | 0 | 0 | 1 |
| **Irinotecan** | Cluster 6 | IRI | CHEMBL481 |  | 586.28 | 8.10 | 7.50 | 319.57 | 278.00 | -5.06 | 3.40 | -21.35 | -7.41 | -5.88 | -3.45 | 0.25 | 0 | 1 | 1 | 1 | 1 |
| **Isoniazid** | Cluster 6 | ISO | CHEMBL64 |  | 137.06 | 11.19 | 5.67 | 279.00 | 171.97 | 0.01 | -0.52 | -9.20 | -4.39 | -5.12 | -3.36 | 0.97 | 1 | 0 | 0 | 0 | 1 |
| **Itraconazole** | Cluster 6 | ITR | CHEMBL64391 | 0.08 | 704.24 | 10.31 | 6.09 | 343.61 | 166.20 | -7.10 | 4.90 | -21.90 | -8.00 | -4.94 | -3.11 | 0.01 | 1 | 1 | 1 | 1 | 1 |
| **Ketocozole** | Cluster 6 | KET | CHEMBL157101 | 1.89 | 530.15 | 7.97 | 6.40 | 301.06 | 145.23 | -3.80 | 3.90 | -19.25 | -0.74 | -4.82 | -3.73 | 0.04 | 1 | 0 | 1 | 0 | 1 |
| **Pirprofen** | Cluster 6 | PIR | CHEMBL188952 | 3.19 | 251.07 | 1.57 | 1.10 | 351.00 | 98.80 | -3.61 | 0.08 | -6.97 | -7.28 | -4.50 | -2.42 | 0.02 | 1 | 1 | 0 | 0 | 1 |
| **Propofol** | Cluster 6 | PRO | CHEMBL526 | 11.8 | 178.14 | 11.09 | 4.39 | 253.85 | 17.10 | -3.16 | 3.10 | -5.63 | -1.70 | -4.74 | -1.27 | 0.02 | 1 | 1 | 0 | 0 | 1 |
| **Valproate** | Cluster 6 | VPA | CHEMBL109 | 6.47 | 144.12 | 5.25 | 5.35 | 219.30 | 125.00 | -1.86 | -0.48 | -5.57 | -2.14 | -4.32 | -1.65 | 0.15 | 1 | 1 | 0 | 0 | 1 |

Variables include normal dose estimated in mmol/day, molecular weight, acid and base dissociation constants (pKa-a and pKa-b), boiling point (BP), melting point (MP), water solubility (WS), distribution coefficient (logD), hydrophobicity (logH), vapor pressure (logVP), Caco-2 cell permeability (Caco2), skin permeability (logKp), and fraction unbound in plasma (FU). Variables labeled as "class" are categorical and include oral bioavailability ≥ 30% (F30), blood-brain barrier penetration (BBB), P-glycoprotein inhibition potential (pgp_inh), P-glycoprotein substrate status (pgp_subs), and human intestinal absorption (HIA). Log-transformed units are indicated where applicable.

**Table S4 Chemical variables examined for the DIFLD causing drugs.**

| **Clusters** | **mmol**  **per**  **day** | **WS*** | **logD** | **pgp**  **inh** | **pgp**  **subs** | **F30** | **Ratio pkaa/**  **pkab** | **HIA** | **MW** | **pKab** | **LogH*** | **BBB** | **FU** |
| --- | --- | --- | --- | --- | --- | --- | --- | --- | --- | --- | --- | --- | --- |
| **Cluster 1-2** | 0.3 | 2.0 | 5.4 | 4.6 | 2.3 |  |  | 1.6 |  |  |  | 0.5 | 0.2 |
| **Cluster 3** | 4.7 | 0.7 | -0.4 |  | 3.2 | 1.6 | 1.6 | 1.6 | 0.6 | 0.6 | 0.6 | 0.6 |  |
| **Cluster 4-5** | 2.7 |  | 2.6 | 2.3 | 3.4 |  | 1.6 |  |  |  | 0.6 | 0.5 |  |
| **Cluster 6** | 5.2 |  | 2.5 | 2.2 |  |  |  |  |  |  | 0.6 |  | 0.4 |

The table displays the fold change in mean values of selected physicochemical variables, detailed in Table S3, for each cluster of steatosis-associated drugs, relative to the mean values of Cluster 0 (non-steatotic compounds). Only variables exhibiting a change of ±50% or greater compared to Cluster 0 are included. Variables marked with an asterisk (*) had negative mean values; for these, fold changes are reported in absolute terms. Color coding is used to indicate the direction of change: red for increases and blue for decreases relative to Cluster 0. The continuous variables include: Normal dose estimated in mmol/day; water solubility (WS); distribution coefficient (logD); P-glycoprotein inhibition potential (pgp_inh); P-glycoprotein substrate status (pgp_subs); oral bioavailability ≥ 30% (F30); ratio of acid to base dissociation constants (pKa-a/pKa-b); human intestinal absorption (HIA); molecular weight (MW); base dissociation constant (pKa_b); hydrophobicity (logH); blood-brain barrier penetration (BBB); and fraction unbound in plasma (FU). Cluster 0 comprises reference (non-steatogenic) drugs. Clusters 1–2 are linked to mild or moderate steatosis and are characterized by low systemic dose, balanced lipophilicity (logD), and enhanced water solubility (WS). Cluster 3, associated with lactic acidosis, includes hydrophilic, low-MW compounds with high oral absorption (HIA) and reduced logD. Clusters 4–5, related to inflammatory phenotypes, show moderate dosing, elevated lipophilicity, and limited BBB permeability. Cluster 6, steatogenic compound with cholestatic features, is defined by marked lipophilicity, low plasma availability (FU), and increased potential to inhibit P-glycoprotein (pgp_inh).

**Table S5: Classification of DIFLD Based on Clinical Phenotypes**

| **Cluster** | **Biochemical Pattern** | **Pathological Features** | **Clinical Implication** | **Monitoring/Management Recommendations** |
| --- | --- | --- | --- | --- |
| 0 | No noticeable biochemical changes | Non reported | Unlikely noticeable clinical effects | No need of monitoring |
| 1 | Minimal/no change in ALT, AST, GGT, bilirubin | Mild, progressive macrovesicular steatosis | Gradual fat accumulation in metabolic patients with risk of chronicity and fibrosis | Long-term monitoring of liver function; consider risk factors (e.g., obesity, diabetes) |
| 2 | Mild ALT & AST elevation; minimal/no change in bilirubin | Moderate steatosis | Risk of progression to steatohepatitis in metabolic patients or liver disease | Regular LFTs; metabolic monitoring (glucose, lipids); adjust treatment in at-risk patients |
| 3 | May have normal or elevated LFTs; ↑ lactate | Mitochondrial dysfunction, lactic acidosis, possible ductular/cholestatic changes, mild inflammation | Risk of acute, severe liver injury and metabolic collapse | Monitor lactate, acid-base balance, LFTs; rapid drug discontinuation if toxicity suspected |
| 4 | Moderate ↑ in ALT, AST, GGT & bilirubin | Macrovesicular steatosis; Early steatohepatitis; | Slow evolution toward fibrosis/cirrhosis if untreated | Regular ALT, AST, ALP, GGT; intervene early; consider biliary disease risk |
| 5 | Marked ↑ in ALT & AST; possible ↑ GGT | Microvesicular steatosis; severe hepatotoxicity an steatohepatitis | Rapid liver injury, especially in patients with alcohol use or liver disease | Frequent LFTs and liver imaging; avoid in high-risk patients; dose adjustment or alternative therapy |
| 6 | Very high ↑ Bilirubin, moderate ALP, ALT & AST | Microvesicular steatosis; Concurrent cholestasis with an inflammatory component | Risk of prolonged cholestasis and liver dysfunction | Monitor bilirubin, ALP closely; act quickly on cholestatic signs; consider safer alternatives |

Proposed classification system for DIFLD, integrating biochemical patterns, histopathological features, clinical implications, and monitoring or management recommendations across seven phenotypic clusters (0–6)
